# Raman Spectroscopy Adds Complementary Detail to the High-Resolution X-Ray Crystal Structure of Photosynthetic PsbP from *Spinacia oleracea*

**DOI:** 10.1371/journal.pone.0046694

**Published:** 2012-10-05

**Authors:** Vladimir Kopecky, Jaroslava Kohoutova, Mikalai Lapkouski, Katerina Hofbauerova, Zofie Sovova, Olga Ettrichova, Sergio González-Pérez, Alexander Dulebo, David Kaftan, Ivana Kuta Smatanova, Jose L. Revuelta, Juan B. Arellano, Jannette Carey, Rüdiger Ettrich

**Affiliations:** 1 Institute of Physics, Faculty of Mathematics and Physics, Charles University in Prague, Prague, Czech Republic; 2 Institute of Nanobiology and Structural Biology, Global Change Research Center, Academy of Sciences of the Czech Republic, Nové Hrady, Czech Republic; 3 Faculty of Fisheries and Protection of Waters, University of South Bohemia, Nové Hrady, Czech Republic; 4 Institute of Microbiology, Academy of Sciences of the Czech Republic, Prague, Czech Republic; 5 Faculty of Sciences, University of South Bohemia, České Budějovice, Czech Republic; 6 Departamento de Estrés Abiótico, Instituto de Recursos Naturales y Agrobiología de Salamanca (IRNASA-CSIC), Salamanca, Spain; 7 Departamento de Microbiología y Genética, Instituto de Biología Funcional y Genómica, Universidad de Salamanca/CSIC, Campus Miguel de Unamuno, Salamanca, Spain; 8 Chemistry Department, Princeton University, Princeton, New Jersey, United States of America; National Institute for Medical Research, United Kingdom

## Abstract

Raman microscopy permits structural analysis of protein crystals *in situ* in hanging drops, allowing for comparison with Raman measurements in solution. Nevertheless, the two methods sometimes reveal subtle differences in structure that are often ascribed to the water layer surrounding the protein. The novel method of drop-coating deposition Raman spectropscopy (DCDR) exploits an intermediate phase that, although nominally “dry,” has been shown to preserve protein structural features present in solution. The potential of this new approach to bridge the structural gap between proteins in solution and in crystals is explored here with extrinsic protein PsbP of photosystem II from *Spinacia oleracea*. In the high-resolution (1.98 Å) x-ray crystal structure of PsbP reported here, several segments of the protein chain are present but unresolved. Analysis of the three kinds of Raman spectra of PsbP suggests that most of the subtle differences can indeed be attributed to the water envelope, which is shown here to have a similar Raman intensity in glassy and crystal states. Using molecular dynamics simulations cross-validated by Raman solution data, two unresolved segments of the PsbP crystal structure were modeled as loops, and the amino terminus was inferred to contain an additional beta segment. The complete PsbP structure was compared with that of the PsbP-like protein CyanoP, which plays a more peripheral role in photosystem II function. The comparison suggests possible interaction surfaces of PsbP with higher-plant photosystem II. This work provides the first complete structural picture of this key protein, and it represents the first systematic comparison of Raman data from solution, glassy, and crystalline states of a protein.

## Introduction

Optical spectroscopy is often used as an alternative to protein structure determination by x-ray crystal diffraction or NMR, although it cannot provide complete atomic-level information about three-dimensional (3D) structure. Among other outstanding qualities, Raman spectroscopy [1] provides a wealth of detailed information about protein structure, especially in combination with molecular modeling [2]. Raman spectroscopy also presents a unique opportunity to study and compare protein samples in different phases, including intact protein crystals directly as they grow in hanging drops [3]. For example, Raman-assisted crystallography [4] couples Raman spectroscopy *in situ* to X-ray crystallography at synchrotrons. The main value of this technique is on-line monitoring during data collection for real-time information on the integrity of protein crystals, such as radiation damage or X-ray induced chemistry (e.g., disulphide bond breakage [4]–[6]). Raman microscopy of protein crystals, on the other hand, can identify differences between the crystal and solution states. These differences have typically been ascribed to the structure of the water layer surrounding the protein in the two states [7]–[10], although the origin of the effects has remained unclear. Methods that can differentiate spectral changes due to effects in the water envelope versus effects on the protein itself are being developed currently [11], [12].

In the present work a new, fast technique of nonresonance Raman microscopy is applied to this problem. The method relies on a drop-coating deposition Raman (DCDR) approach [11], [13] based on a so-called coffee-ring effect [14] that produces samples of biomolecules in a glassy state [2] that can be considered as intermediate between solutions and crystalline solids. These nominally solid-phase samples have a low water content, yet they preserve the solution structural characteristics of biomolecules [15]. In particular, DCDR analyses have shown that the vibrational modes of proteins in the glassy state are more similar to those in solution than to those in crystals [12], [13], suggesting that the differences detected by Raman between crystals and DCDR deposits reflect crystalline order rather than solvent content. The DCDR method enables nondestructive measurements on biomolecules with typical concentrations in the original solution down to ∼1 µM [11], [15]; 0.5 µL of 0.01 mg/mL protein solution is sufficient.

As an example demonstrating the effectiveness of this new approach that connects x-ray crystal diffraction and molecular modeling with Raman spectroscopy of solutions, glassy states, and crystals, the present work addresses the high-resolution 3D structure of the extrinsic protein PsbP of photosystem II (PSII) from spinach chloroplasts, *Spinacia oleracea*. The light-driven redox reaction at the catalytic centre, the oxygen-evolving complex (OEC), releases molecular oxygen as a by-product on the lumenal side of PSII [16]. The higher plant OEC consists of an inorganic Mn_4_-oxo-Ca cluster that is apparently stabilized by extrinsic proteins PsbO, PsbP, PsbQ, and PsbR. PsbP and PsbQ proteins apparently maintain the ionic environment during water oxidation [17], [18], and they control access by substrates and products, in particular limiting access by reductants other than water [19]. In transgenic tobacco plants lacking PsbP the catalytic cluster is unstable, indicating a requirement for PsbP to support PSII function *in vivo* [20]. Thus, the regulation and full dynamics of PSII in higher plant thylakoids is clearly dependent on its interactions with these proteins [21]–[23].

The only structural models available to date for higher-plant PSII are from low-resolution electron microscopy [24]–[26], providing limited information about the interactions of these key peripheral proteins. The recently determined 3D x-ray crystal structure of a cyanobacterial PSII has notably improved upon earlier partial structures of the bacterial complex [27]–[30], but provides no clues to the possible arrangement of PsbP and PsbQ in higher-plant PSII because these proteins are absent from cyanobacteria [31]. High-resolution x-ray crystal structures are known for spinach PsbQ [32] and for tobacco PsbP [33], and for the PsbP-like protein CyanoP from *Thermosynechococcus elongatus* [34], but their interaction sites on PSII have remained elusive. CyanoP homologs are found even in organisms lacking thylakoids, indicating a fundamentally different role despite a structure virtually identical to that of tobacco PsbP. The tobacco PsbP structure presented three unresolved chain segments, one of which was the N-terminus where 15 residues were missing due to partial degradation [33]. This region of the protein is implicated in PsbP function because a 15-residue N-terminal deletion binds to PSII but does not activate oxygen evolution [21].

**Figure 1:**
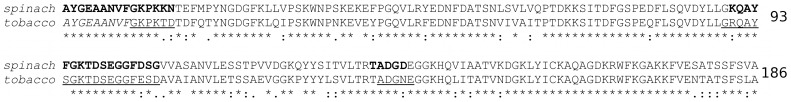
Pair alignment of spinach and tobacco PsbP sequences. Sequences are numbered starting with 1 at the first residue of the mature protein. Asterisks mark identities (149 of 186 residues, 78%). Residues present in the crystalline protein but unresolved in the electron density are bold (spinach) or underlined (tobacco); residues 1 to 9 of the tobacco structure were missing due to partial degradation [33].

Crystals of full-length, mature spinach PsbP suitable for high-resolution structure determination have been reported previously [35]. The analysis of diffraction data to 1.98 Å on such crystals is now reported in the present work. The structure shares with tobacco PsbP the same internal regions that are unresolved in the electron density (Figure 1), and, although the N-terminus is intact in the spinach protein, it too is not resolved. In an effort to achieve as complete a structure as possible for spinach PsbP, and to shed light on its potential interaction surface with PSII, Raman analysis in solution, glassy, and crystalline states of the protein is combined with *de novo* modeling of the unresolved internal regions. Comparison of the resulting structure with that of CyanoP [34] reveals regions of structural difference that may reflect the differing roles of these proteins with respect to PSII function.

## Results and Discussion

### PsbP x-ray crystal structure

**Figure 2:**
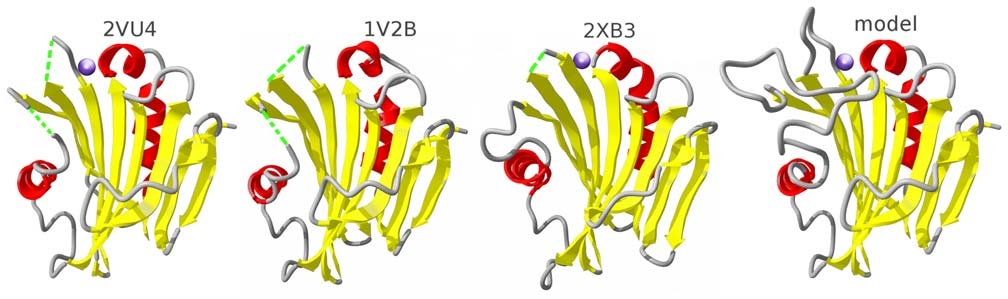
PsbP-family structures. X-ray structures of spinach PsbP, tobacco PsbP, and CyanoP (left to right, identified by PDB ID); the fourth structure is the loop-modeled spinach PsbP reported here. The cartoon identifies secondary structure as β-strand, yellow; helix, red; loop or turn, grey.

As described in Methods, the recombinant PsbP protein from spinach was crystallized and the crystals used for x-ray analysis under conditions slightly different from those reported previously [35] in order to control crystal size for synchrotron analysis. Figure 2A shows the protein structural model resulting from analysis of diffraction data extending to 1.98 Å; statistics from the structural analysis are presented in Table 1. The data are deposited with PDB accession code 2vu4. Although purified spinach PsbP protein shows no degradation products by analytical SDS gel electrophoresis and full-length protein is recovered from dissolved crystals as reported already [35], the electron density is not resolved in the N-terminal region (residues 1–15) and in two internal regions (residues 90–107 and 135–139), as in the structure of tobacco PsbP (PDB ID 1V2B [33]; Figure 1). One Zn^2+^ ion is coordinated by spinach PsbP *via* the sidechains of His144 and Asp165, corresponding to His142 and Glu163 of CyanoP that coordinate a Zn^2+^ ion. It has been suggested [36] that one or more of the observed Zn^2+^-binding sites might be sites for the physiologically relevant ions Mn^2+^ or Ca^2+^. If so, this common Zn^2+^ site might mark a functionally important surface of the protein.

**Table 1:** X-ray data collection and refinement statistics.

**Data collection**	
Space group	*P*2_1_2_1_2_1_
Cell dimensions (Å)	*a* = 36.77, *b* = 45.57, *c* = 82.50
Resolution limit (Å)	45.50–1.98 (2.09–1.98)
Completeness (%)	89.7 (92.4)
Redundancy	2.8 (3.8)
Mean *I*/σ*I*	11.1 (1.8)
*R*_merge_ (%)	11.2 (50.6)
**Refinement**	
Resolution range (Å)	30.0-1.98
No. of reflections	8009
*R*_work_/*R*_free_ (%)	18.1/23.3
No. of atoms	
Protein	1138
Zn^2+^	1
Water	34
*B*-factors	
Protein	25.90
Zn^2+^	35.20
Water	41.99
R.m.s. deviations	
Bond lengths (Å)	0.024
Bond angles (°)	2.07

Values in parentheses are for the highest-resolution shell.

This second structure of a higher-plant PsbP protein offers the first chance for a detailed structural comparison to define common and variable features. In fact the three-dimensional structures of spinach and tobacco PsbP hardly differ, overlaying with an RMSD of 1.07 Å for all 148 resolved C_α_-atoms, consistent with their sequence identity of 78% for the 186 residues of the mature protein (Figure 1). The only region of structural difference, at residues 164–173, appears to be related to crystallization. Although the sequence of this segment, GDKRWF**K**GAK, is identical in the two proteins, their secondary structures differ slightly, probably due to an interaction in the tobacco structure between Lys170 (bold underlined) and a solvent sulfate ion. The N-terminal end of the segment is anchored in the spinach PsbP structure by the interaction of Asp165 with the Zn^2+^ ion; short α-helix C (GDKRWF) is followed by a two-residue H-bonded turn (**K**G), and the last two residues (AK) are part of the first turn of helix D. Tobacco PsbP shows one turn of 3_10_-helix (GDKR) followed by a large H-bonded turn (WF**K**GAK) leading to helix D. The sulfate ion attracts the side chain of Lys170 in the opposite direction as in the spinach structure, probably causing this difference. Although sulfate was also present at high concentration in the spinach PsbP crystals, their different space group and hence different crystal contacts may explain the absence of a sulfate ion.

### Raman spectroscopy

**Figure 3:**
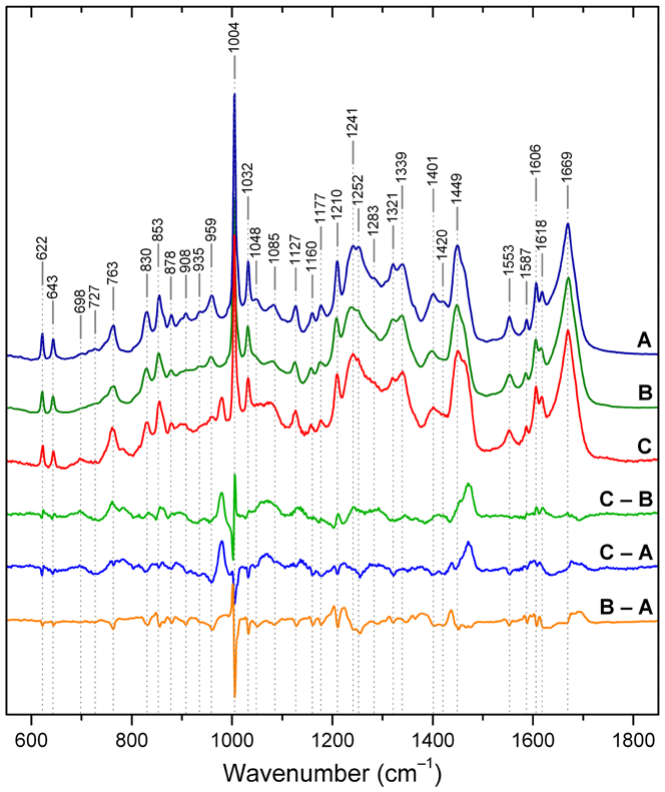
Raman spectra of spinach PsbP. Spectra acquired on samples of protein in solution (**A**), DCDR glassy deposit (**B**), and crystal (**C**). Difference spectra are depicted below the figure: DCDR minus solution (**B – A**); crystal minus solution (**C – A**); crystal minus DCDR (**C – B**). The frequencies indicated by vertical dashed lines mark band positions in solution. Band assignments are presented in Table 2.

As described in Methods, spinach PsbP was crystallized under conditions slightly different from those used for x-ray structure determination in order to obtain the large crystals required for Raman crystal analysis. For spinach PsbP in crystal and glassy states Figure 3 shows the Raman water vibration region, which is centered around 3300 cm^−1^. In pure water the most intense band in this region has a maximum at ∼3400 cm^−1^. In the crystal sample this water band is of slightly higher intensity than in the DCDR sample, in agreement with theoretical calculations [15]. In solution samples this water band is at least two orders of magnitude stronger, obscuring the nearby protein bands (data not shown). Thus, relative to the intensity of the water band in solution samples, both crystal and DCDR states of PsbP have very similar water content, although the shapes of the bands differ.

**Figure 4:**
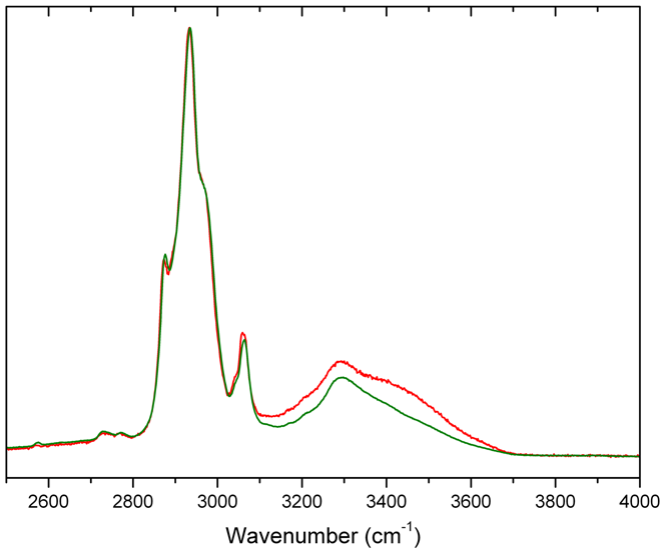
Water vibration region of spinach PsbP Raman spectra. Spectra were acquired on samples of protein in DCDR (green) and crystal (red). The spectra are centered on the most intense Raman water band with maximum at ∼3400 cm^−1^.

The Raman spectra of spinach PsbP in crystal, solution, and glassy states are depicted in Figure 4. The three spectra are highly similar, with no substantial overall difference in the sharpness of peaks in the three spectra, indicating no general or widespread difference in stiffness of vibrational modes in the three states. All three pairwise difference spectra are shown below the spectra. The glassy minus solution difference spectrum (B – A) is almost featureless, consistent with previous evidence that protein structure in these two states is similar [11], [12]. Thus, despite the nominally “dry” condition and different composition of the glassy samples (see Methods), the protein maintains not only its overall secondary and tertiary structure, but also the structural features characteristic of the solution state. The few well-defined small peaks in the B – A difference spectrum occur adjacent to, rather than coincident with, bands that are assigned to known modes in the solution state, indicating minor differences in frequency that reflect changes in local environment.

The crystal minus glassy (C – B) and crystal minus solution (C – A) difference spectra are highly similar to each other, and both display a small number of prominent features not observed in the glassy minus solution difference spectrum (B – A). These results indicate that the crystal state differs similarly, yet surprisingly little, from both the solution and glassy states. The prominent difference peak at 1470 cm^−1^ arises from the broad, intense band centered at ∼1450 cm^−1^ that is ascribed to bending vibrations of CH_2_ and CH_3_ groups [36], [37], [38]. In the solution and glassy states only a shoulder appears at ∼1470 cm^−1^ (see Figure 4, curve A and B). In the crystal state the high-frequency edge of the broad band splits more distinctly into a band of intensity almost equal to that of the main peak. This change indicates that a substantial subset of the methyl and methylene groups respond to the crystalline environment by populating a narrower distribution of vibrational modes. As these groups belong to nearly every protein residue type, the result suggests a widespread response over the protein. This response may reflect that in the solid states the amount of water in the hydrophobic hydration layer is limited, and may in turn limit the vibrational modes that can be sampled. A new band at 978 cm^−1^, observable only in the crystal, is of unknown origin. No intensity is present at this frequency in the spectrum of the crystallizing buffer (Figure S1).

Assignments of Raman bands are given in Table 2. Representative modes indicate the nature of the differences among the three states. Several bands associated with aromatic residues are sharper in the solution spectrum than in the glassy or crystal spectra, giving rise to coincident peaks that are negative in the crystal minus glassy (C – B) and crystal minus solution (C – A) difference spectra. The tryptophan band at 1553 cm^−1^ has been correlated with the absolute value of the torsional angle |χ^2,1^| of C_2_-C_3_-C_β_-C_α_ [37]; the observed frequency corresponds to |χ^2,1^| torsions close to 100° for both Trp residues. This band is sharper in the solution spectrum than in the glassy or crystal spectra. This change indicates that in solution one or both Trp residues populate a narrower distribution of conformations than in the two solid states. Similarly, Lys contributes to several low-intensity bands at 1085, 1048, 959, 935 and 878 cm^−1^ [38], [39], all of which are broader and less intense in the crystal spectrum and become more intense and sharper in the solution spectrum. Residues that can form hydrogen bonds are likely to populate a broader distribution of conformers in the relatively inflexible solid states than in solution states where flexibility allows better optimization of bond geometry.

**Table 2:** Assignment of the Raman bands of spinach PsbP.

Frequency (cm^−1^)	Assignment
622	Phe [38]
643	Tyr [38]
698	υC–S P_N_, P_H_ [40]
727	υC–S P_C_ [40]
763	Trp W18 [38], [40]
830	Tyr doublet [38], [39], [40]
855	Tyr doublet [38], [39], [40]
863	Tyr, Ile [38], [39]
878	Lys [38], [39]
908	Ala [38], [39]
935	α-helix, Lys, Val, Leu [38], [39]
959	Lys, Leu [38], [39]
992	Ile shoulder [38], [39]
1004	Phe [38], [39], [40]
1007	Trp shoulder [38], [39], [40]
1032	Phe [38], [39], [40]
1048	Lys, Ala, Phe [38], [39]
1085	Lys, Phe [38], [39]
1127	Ile, Val, Leu, Trp [38], [39]
1160	Ile, Val [38], [39]
1177	Tyr, Phe [38], [39]
1210	Phe, Tyr, Trp [38], [39]
1241	amide III [38], [39]
1252	amide III, Trp [38], [39]
1283	amide III, Tyr [38], [39]
1321	Tyr, Trp [38], [39]
1339	Trp doublet [38], [39], [40]
1363	Trp doublet [38], [39], [40]
1401	υCO_2_^−^ of Asp, Glu [41]
1420	Trp [38], [39]
1449	δCH_2_, δCH_3_, Lys, Ile, Leu [38], [39]
1463	δCH_2_, Ala, Ile, Val, Leu, Trp, Tyr [38], [39]
1553	Trp W3 [37]
1587	Trp, Phe [38], [39]
1606	Phe, Tyr [38], [39]
1618	Tyr, Trp [38], [39]
1669	amide I [38], [39], [40]

### Raman analysis of secondary structure content

Secondary structure content was estimated from the three sets of Raman data by analyzing the amide I band using the pattern-recognition least-squares method (LSA) [42] and two reference intensity profile methods (3-RIP and 4-RIP) [43]. The results are shown in Table 3. The three methods have slightly different strengths in secondary structure estimation due to different reference sets as well as due to different mathematical treatments. Within their errors, the three secondary structure estimations taken together show no trend among the three protein states. In rough numbers the three methods agree that the secondary structure content of PsbP in all three states is ∼20% helices and ∼50% β-strands; the three estimation methods differ greatly in their assignment of the remaining ∼30% to β-turns or disordered structure, reflecting a well-known weakness [44]. In the x-ray crystal structure with 148 residues resolved, 26 residues are in helical conformation, 75 in β-strands, 23 in turns, and 24 unordered. Irrespective of the differences among the estimates, and their known limitations, the minimum number of residues in β-sheet according to Raman secondary structure content estimation is 87. The number of residues in β-strands in the x-ray crystal structure is 75, indicating that the unresolved segments probably contribute additional residues to β-strands or sheets.

**Table 3:** Secondary structure content of PsbP.

	SOLUTION			DCDR DEPOSIT			PROTEIN CRYSTAL			
Structure	LSA	3-RIP	4-RIP	LSA	3-RIP	4-RIP	LSA	3-RIP	4-RIP	Model
α-helix	25±5 (48)	23±3 (44)	19±3 (36	19±5 (36)	17±3 (32)	16±3 (30)	19±5 (36)	18±3 (34)	16±3 (30)	17 (32)
α-ordered	18±4 (34		18±3 (34)	13±4 (25)		10±3 (19)	13±4 (25)		12±3 (23)	
α-disordered	7±4 (13)		1±3 (2)	5±4 (10)		6±3 (11)	6±4 (11)		3±3 (6)	
β-sheet	46±4 (87)	50±3 (95)	49±3 (93)	51±4 (97)	58±3 (110)	57±3 (108)	51±4 (97)	57±3 (108)	56±3 (106)	40 (75)
β-turn	19±2 (36)		9 (16)	20±2 (38)		9 (16)	20±2 (38)		9 (16)	8 (15)
Unordered	10±2 (19)	27±3 (51)	32±3 (61)	10±2 (19)	25±3 (48)	27±3 (51)	10±2 (19)	26±3 (49)	28±3 (53)	35 (67)

The amide I band was analyzed from Raman spectra acquired on protein samples in solution, glassy state (DCDR), and crystals. Spectra were deconvoluted using the pattern recognition least-squares method (LSA) [38] and two reference intensity profile methods (3-RIP and 4-RIP) [39]. Secondary structure content is given as % of residues ± standard deviation calculated from the standard deviations for each respective reference set. All % values are based on the full sequence of 190 residues; the number of residues in each secondary structure type is given in parentheses. The 4-RIP method does not normalize to 100%. The categories α-ordered and α-disordered structures reflect helix mobility. In the model, the 15 native and 4 remaining His-tag residues were assigned as unordered, and added to the 48 residues observed in that conformation.

Despite the rough agreement of the estimations for the three different states, detailed analysis of the amide I and amide III bands indicates small systematic differences. The region of the amide I band is extremely sensitive both to changes in secondary structure content, as well as to aggregation and oligomerization [41]. The absence of a difference peak in the crystal minus DCDR (C – B) spectrum excludes the presence of aggregated protein in the DCDR sample. In both crystal minus solution (C – A) and DCDR minus solution (B – A) spectra in the region of amide I band, the difference is positive at ∼1670–1712 cm^−1^, reflecting higher content of β-structures. In contrast, the negative band at 1630–1650 cm^−1^ reflects lower content of α-helical structures. These changes in turn agree well with those in the region of amide III, i.e., positive at 1213–1234 cm^−1^ and negative at 1235–1300 cm^−1^. The band at ∼935–940 cm^−1^, which also indicates the amount of α-helical structure [39], agrees with the changes in amide I and amide III bands by showing a negative value. These results indicate that the solution structure is characterized by a slight increase in α-helix and an decrease in β-sheet structure, while the differences between the crystal structure and the DCDR sample are negligibly small, if any, as can be also seen from the difference (C – B) in Figure 4.

Although in other respects it reflects characteristics of the solution structure, the DCDR sample is closer to the crystal state with respect to secondary structure estimation. As the secondary structure differences between the crystal state and the glassy state are negligibly small, the small differences between solution and crystal states are not likely due to effects of the crystal lattice, but rather reflect the low water content in both glassy and crystal states. DCDR spectroscopy could thus serve as an easy-to-use method to distinguish between structural changes due to crystal contacts and those coming from the environment, in cases where crystal and solution structures do not agree.

### Molecular modeling

Crystals of spinach and tobacco PsbP have different space groups and crystal contacts, suggesting that the three regions that are unresolved in both structures might be intrinsically flexible rather than affected by common crystal lattice features. The N-terminal region and the longer of the two internal loops are also missing from the x-ray crystal structure of CyanoP, indicating that these segments are also unresolved in a third set of space groups and crystal contacts. In spinach PsbP the endpoints flanking each missing internal segment are well-defined by the electron density, limiting the chain excursions taken by the adjacent missing residues. These factors support the use of molecular dynamics and established loop-modeling methods to define the 3D structure of these internal segments in an effort to fill in as much as possible the structural picture of PsbP and its interactions with PSII. The N-terminal region is not suited for modeling by these methods, which require anchoring residues flanking the missing segment on both sides.

**Figure 5:**
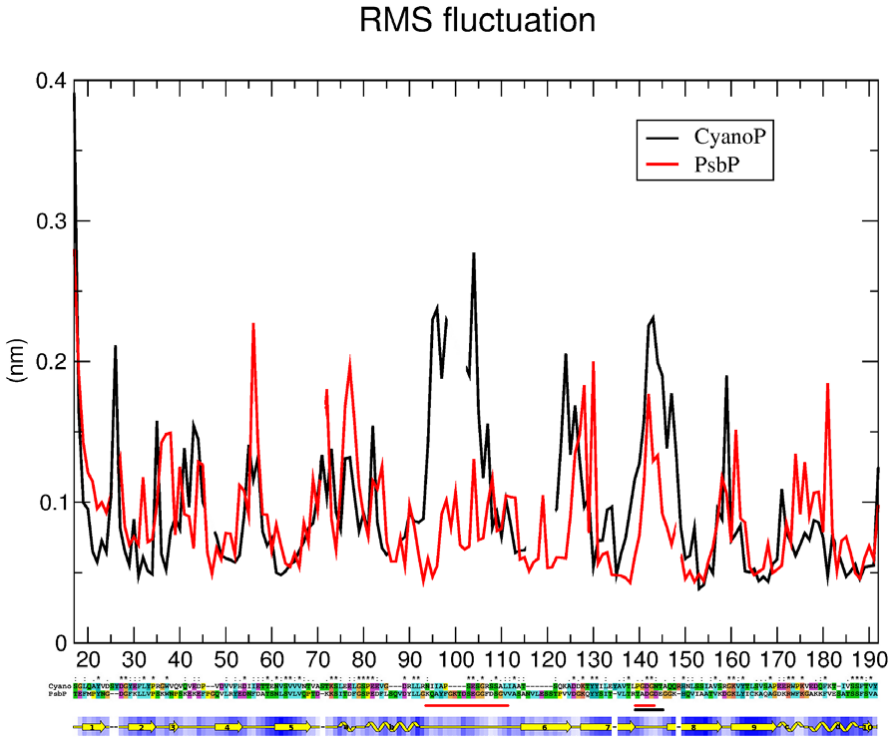
Modeling of spinach PsbP and cyanoP. **Top**, root mean square fluctuation (y-axis, nm) of each C_α_ atom (x-axis, residue number) during the production phase of molecular dynamics simulations of spinach PsbP (red) and CyanoP (black). **Middle**, pair alignment of spinach PsbP and CyanoP sequences. Sequences begin with the first residue resolved at the N-terminus of the respective crystal structures. The two sequences were aligned and colored by Clustal X [45]. Identical residues are marked with an asterisk, those with high similarity with a colon, and those with lower similarity with one dot. The two red bars mark the large and small loops in PsbP, the black bar the small loop in CyanoP. **Bottom**, secondary structure (yellow) and water accessibility (blue) of spinach PsbP based on Procheck [46]. Strands are represented as arrows, helices as folded tape, irregular regions as a line, gaps as a dash. Accessibility is shaded from white (fully accessible) to dark (fully buried).

Starting with the deposited x-ray crystal structure of spinach PsbP, PDB accession code 2vu4, modeling of the two internal unresolved segments, residues 90–107 and 135 to 139, was performed using available programs as described in Methods. The best model judged as described in Methods was used as the input for molecular dynamics simulation as described in Methods. Simulations of the water-solvated protein model were run for 15 ns; the system reached equilibrium at about 10 ns, as judged from the absence of further trends in the RMSD of the simulated structure relative to the starting structure (data not shown). Outside the modeled segments the simulations led to only one minor alteration as compared with the crystal structure, in the region of structural difference between spinach and tobacco PsbP. Helices C and D of spinach PsbP show extremely stable behavior as indicated by RMSF analysis (Figure 5), but Lys170 adopts a helical conformation, extending helix C and leaving Gly171 as the only turn residue.

**Figure 6:**
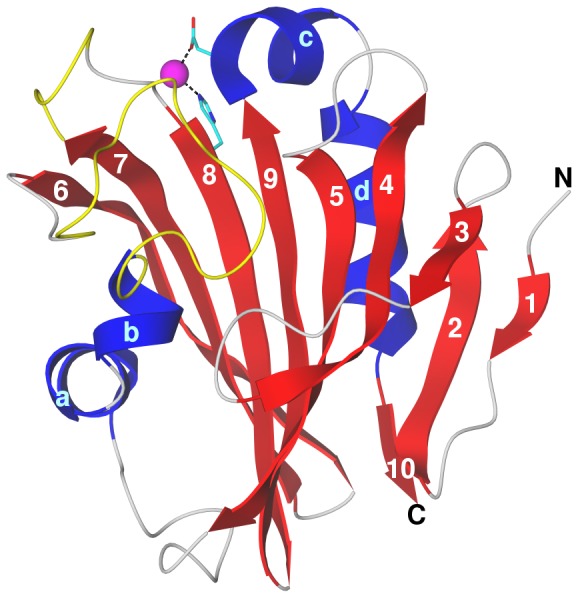
Modeled structure of spinach PsbP. The structure is shown after 15 ns of molecular dynamics at 300 K. Secondary structure elements are indicated: strands, numbered blue; helices, lettered red; irregular, grey; modeled internal loops, yellow (longer loop, residues 90 to 107 between helix b and strand 6; shorter loop, residues 135 to 139 between strands 7 and 8). The Zn^2+^ ion (magenta sphere) is coordinated by Asp165 carboxylate and His144 imidazole (side chains in atomic colors with cyan carbons). The termini are labeled; the N-terminus is that of the crystal structure starting at residue 16.

The long segment between residues 90 and 107 displays surprisingly limited flexibility considering its length and sequence (KQAYFGKTDSEGGFDSGV), with almost half its residues having a statistical propensity for turn-like structures [47]. However, several hydrogen bonds that persist during the entire equilibrated phase limit the excursions of this segment (Gln91–Thr93, Ser99–Gly102, and Phe103–Gly106). During the simulation this segment remains packed against the N-terminal end of the central β-strand (strand 9 in Figure 6), with fluctuations similar to those in random coil conformation elsewhere in the protein. The segment between residues 135 and 139 enjoys slightly greater flexibility more typical of very short loops, making this segment of PsbP the most flexible among loops of 5 or more residues. One persistent hydrogen bond forms within this segment, Thr135–Asp137. The two loops point toward each other, with a persistent hydrogen bond formed between Gly101–Thr135. As Thr135 is adjacent to the C-terminus of strand 9, this contact serves to anchor the center of the long loop to a residue with very limited flexibility, constraining the motion of the long loop much more than that of the short loop.

To further probe potential functionally relevant features of the modeled loop regions, the modeled structure was compared with the crystal structure of CyanoP. CyanoP differs fundamentally from PsbP in its interaction with PSII because the binding site for PsbP in cyanobacterial PSII is occupied instead by PsbU and PsbV [31]. Therefore, structural differences between CyanoP and PsbP might be expected in regions involved in binding of PsbP to PSII. Starting from the deposited crystal structure (PDB accession code 2xb3) CyanoP was solvated and simulated using the same conditions as for the PsbP model, with the unresolved loop added by loop modeling similarly as for PsbP. The root mean square fluctuation profile of the two proteins is very similar (Figure 5). The only major difference is that both modeled loops of PsbP have lower fluctuations than the corresponding loops of CyanoP, where the large loop is significantly shorter and extremely flexible. This difference between PsbP and CyanoP in the behavior of these surface loops is consistent with a role for this region of PsbP in binding to PSII.

### Conclusions

The finding that the large modeled loop in spinach PsbP has very limited flexibility for its length is unexpected considering it is not resolved in any of the three available structures. It differs significantly not only in size but also in its dynamic behavior from the corresponding loop of CyanoP. Because it is the only site of major structural difference between the two proteins, these findings suggest that the large loop in PsbP may be involved in the interaction of PsbP with PSII, as these interactions must differ in cyanobacteria and higher plants. The x-ray crystal structure of PsbP, as well as the modeled structure, shows 75 residues in β-conformation, substantially less than expected from Raman measurements in solution, where at least 87 residues are predicted in β-conformation. The only remaining missing segment of the protein after modeling is the N-terminal segment of 15 residues. Therefore it is likely that the N-terminal region contains not entirely random coil structure, but that ∼12 or more residues adopt a β-conformation and are undetected in the electron density due to either static or dynamic disorder in the crystal.

## Materials and Methods

### Preparation of PsbP protein

The His-tagged recombinant PsbP protein of PSII from *Spinacia oleracea* was overexpressed in *E. coli* BL21(DE3)pLysS cells transformed with plasmid pJR3133 and purified as described previously [30]. Four residues remain on the N-terminus from the His tag. PsbP in 20 mM bis-Tris pH 6.0 (buffer A) was concentrated to a final concentration of 15 mg/mL for crystallization and spectroscopic analysis using centrifugal filter devices (Amicon Ultra 10,000 MWCO; Millipore, Billerica, MA), and then diluted into the conditions required for each analysis as indicated below.

### Crystallization of PsbP protein

The PsbP protein was crystallized using the sitting-drop vapor-diffusion technique as described previously [30] but with the following slightly different conditions: PsbP protein (15 mg/mL) in buffer A was mixed in 1∶1 ratio with reservoir solution containing 16% PEG monomethylether (MME) 550, 0.1 M Tris-HCl, pH 7.5, 10 mM ZnSO_4_, and equilibrated at 288 K for three days. PsbP crystals for Raman microscopy were prepared using the hanging-drop vapor-diffusion technique under conditions that are identical to these except at pH 7.0, which results in the larger crystals that are required for this method.

### X-ray data collection and structure determination

Crystals were soaked in mother liquor containing 25% (w/v) glycerol prior to flash-cooling in liquid nitrogen. The diffraction data set was collected at a fixed wavelength of 0.933 Å at ESRF beam line ID14-2 using an ADSC Q4 CCD detector, a crystal-to-detector distance of 160 mm, and 1° oscillation angle. The total of 180 diffraction images were collected and integrated to 1.98 Å resolution with MOSFLM [48]. Data scaling, merging and intensity conversion to structure-factor amplitudes were carried out with SCALA and TRUNCATE from the CCP4 program package [49]. The crystal data and a summary of the data-collection statistics are listed in Table 3. The crystals belong to space group P2_1_2_1_2_1_, with cell dimensions *a* = 36.77 Å, *b* = 45.57 Å, *c* = 82.50 Å and contain one molecule in the asymmetric unit, with Matthews coefficient [50] 1.74 Å^3^·Da^−1^ indicating a solvent content of 30%. Refinement was carried out in REFMAC5 [51] from the CCP4 suite and model building was performed in Coot [52]. The structure was solved by molecular replacement with MOLREP [53] in CCP4 using the structure of *Nicotiana tabacum* PsbP protein (PDB: 1v2b [29]) as a search model. R_work_ and R_free_ for the final model are 18.1% and 23.3%, respectively. The model was analysed using PROCHECK [46].

### Raman spectroscopy in solution

Raman spectra of aqueous solutions of PsbP were recorded in a standard 90° geometry on a multichannel instrument based on Spex 270M single spectrograph with an 1800 grooves/mm grating (Jobin-Yvon), a holographic notch-plus filter (Kaiser Optical Systems) and a liquid nitrogen cooled CCD detection system (Princeton Instruments) having 1340 pixels along the dispersion axis. The spectral resolution was ∼5 cm^−1^. Spectra were averaged from 300 exposures of 120 s each to produce the traces of highest quality. Samples in a capillary micro-cell (10 µL inner volume) were excited with 532.2 nm line (100 mW of radiant power at the sample) of NdYAG laser (Verdi 2, Coherent) and kept at 277 K during all experiments using an external water bath (Neslab). The wavenumber scale was calibrated with neon glow-lamp lines, thus Raman frequencies of well-resolved bands are accurate to ±0.5 cm^−1^.

### DCDR spectroscopy

PsbP in buffer A was diluted 10-fold in deionized distilled water (18 MΩ), and 4 µL were dialyzed against deionized distilled water for 35 minutes using 0.025 µm VSWP filters (Millipore, 13 mm). A 2 µL volume of the resulting solution, with a PsbP concentration of approximately 1.37 mg/mL, was immediately deposited on a standard DCDR substrate SpectRIM™ (Tienta Sciences) consisting of a polished stainless steel plate coated with a thin layer of Teflon [8]. After drying in air at room temperature (∼20 minutes), Raman spectra were collected from “coffee rings” [10] of former droplets using the Raman microspectrometer as described [9]. The DCDR spectra of the protein in buffer A were measured as well (data not shown). The differences in the spectra were negligible indicating that the protein adopts the same fold under both conditions. The advantage of the dialyzed sample is to eliminate the need of further manipulation of spectra by buffer subtraction.

### Raman microscopy

Raman microscopic measurements were performed using an HR800 Raman microspectrometer (Horiba Jobin Yvon) with 514.53 nm Ar-ion excitation laser (Melles Griot). A 50× microscope objective (N.A. 0.75, Olympus) was used to focus the 5 mW excitation laser to a diameter ∼1.5 µm on the sample, and the spectra were integrated for 600 s. Spectra were collected using a 600 grooves/mm grating and liquid-nitrogen cooled CCD detector (1024×256 pixels, Symphony). The spectrometer was calibrated using band of Si-vibrations at 520.7 cm^−1^; the wavenumber scale was calibrated with neon glow-lamp lines (thus the frequencies of well-resolved bands are accurate to ±0.5 cm^−1^). The spectral resolution was ∼5 cm^−1^ (thus, the spectrometer's slits were adjusted to obtain approximately the same spectral resolution as on the Raman spectrometer used for measurements of PsbP in solution). Protein crystals were measured at room temperature directly in hanging drops in crystallization boxes on the same Raman device using a 50× microscope objective with long working distance (N.A. 0.55, f = 180, Olympus).

### Raman spectra treatment

DCDR spectra are presented directly as they were measured. A buffer blank was subtracted from spectra of PsbP in aqueous solution. For PsbP protein crystals a spectrum collected in the surrounding solution was subtracted from the spectrum of the crystals; the crystal buffer spectrum is shown in Figure S1. Spectral intensities were normalized on amide I band. For the figure comparing the three states the DCDR spectrum was taken as a reference and the spectra of crystal and aqueous solution were fit on it together with polynomial correction (5^th^ grade) of the background.

### Loop modeling and molecular dynamics

The high resolution X-ray structure of spinach PsbP (PDB ID 2vu4) and of CyanoP (PDB ID 2xb3) were used as templates for loop modeling. Bioinformatics methods were used to model the loop regions missing from the electron density, residues 132 to 138 of CyanoP, and residues 90 to 107 and 135 to 139 of spinach PsbP. Ten homology models of PsbP and PsbP-like proteins comprising all non-hydrogen atoms were generated using the Modeller software package [54]. Input sequence alignments were made manually and are shown in Figure 5. The best model for each protein was chosen based on the Modeller objective function [2] and on stereochemical g-factors and the distribution of Ramachandran angles obtained by the program Procheck [53]. Each model was then used as the input for molecular dynamics simulation using the Gromacs 4.0.5 software package [55], [56].

Each protein was solvated in TIP3P water and each system was neutralized by addition of counterions. Weak temperature and pressure coupling [57] were employed (coupling constants 0.1 ps), with the protein and solvent atoms having separate baths maintained at 300 K, and pressure maintained at 1 bar with a compressibility of 4.6*1025/bar. Simulations employed the OPLS force field [58]. Electrostatics was evaluated using the particle-mesh Ewald method [59] with a cutoff of 0.9 nm, and van der Waals forces were evaluated with a Lennard-Jones potential having a 1.4 nm cutoff. Virtual-site hydrogens [60] were employed to increase calculation speed by allowing for time steps of 5 fs. Bond lengths were constrained using LINCS [61]. The neighbor search list was updated every 20 fs. The solvated system was first energy minimized using steepest descent and the solvent was allowed to relax for 250 ps while keeping the protein restrained. Initial Boltzmann-weighted velocities were generated randomly and the system was further equilibrated for 500 ps before initiating molecular dynamics production runs without constraints. For PsbP the helix preceding the longer loop did not maintain a hydrogen bond network in the early stages of the simulation, which allowed it to start unraveling and interacting with other parts of the protein. To allow the modeled loops to equilibrate within the context of the crystal structure, positional restraints were applied for the first 4 ns, followed by the fully unrestrained production run extending through 65 ns.

### Protein Data Bank accession number

The atomic coordinates and experimental data (code 2vu4) have been deposited in the Protein Data Bank (www.wwpdb.org). The complete modeled structure of PsbP protein is available upon request.

## Supporting Information

Figure S1 **DCDR spectrum of the crystallizing buffer for PsbP protein used in Raman crystallography.** Buffer A was mixed in a 1∶1 ratio with crystallization reservoir solution containing 16% PEG 550 MME, 0.1 M Tris-HCl pH 7.0, 10 mM ZnSO_4_.
(TIF)
