# Supplementary figures and images for: Raman Spectroscopy Adds Complementary Detail to the High-Resolution X-Ray Crystal Structure of Photosynthetic PsbP from Spinacia oleracea
Source: PLoS One. 2012 Oct 5;7(10):e46694. doi: 10.1371/journal.pone.0046694 (PMC3465285; doi:10.1371/journal.pone.0046694)

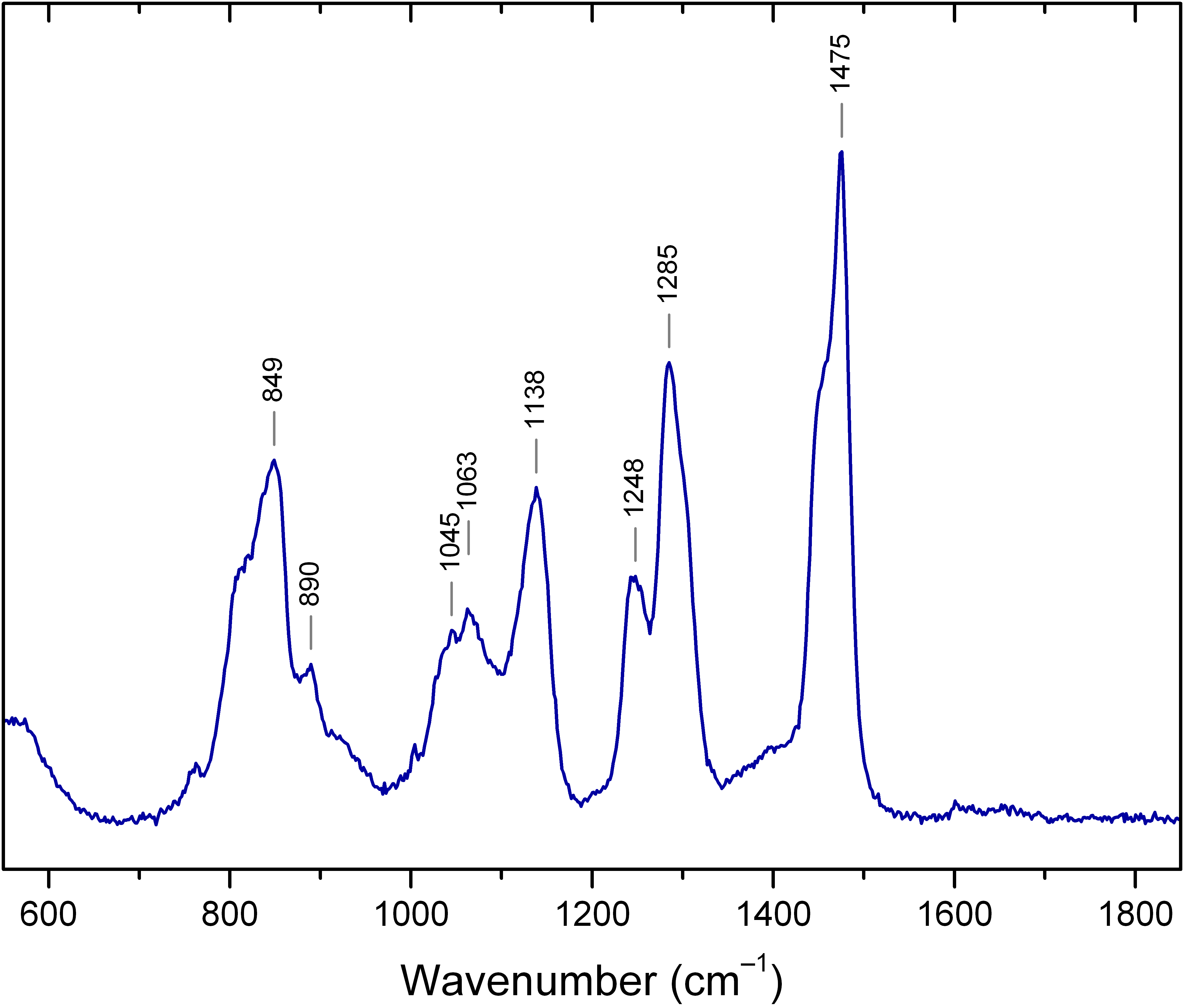

Supplement: Figure S1 — DCDR spectrum of the crystallizing buffer for PsbP protein used in Raman crystallography. Buffer A was mixed in a 1∶1 ratio with crystallization reservoir solution containing 16% PEG 550 MME, 0.1 M Tris-HCl pH 7.0, 10 mM ZnSO4. (TIF) [file pone.0046694.s001.tif]
